# Physical Rehabilitation Crucial in Motor Axonal Neuropathy Following Organophosphorus Poisoning: A Case Study

**DOI:** 10.7759/cureus.54145

**Published:** 2024-02-13

**Authors:** Alfiza Khan, Nikita H Seth, H V Sharath

**Affiliations:** 1 Department of Paediatric Physiotherapy, Ravi Nair Physiotherapy College, Datta Meghe Institute of Higher Education and Research, Wardha, IND; 2 Department of Neuro Physiotherapy, Ravi Nair Physiotherapy College, Datta Meghe Institute of Higher Education and Research, Wardha, IND

**Keywords:** physiotherapy, case report, acute organophosphate exposure, exercises, physiotherapy rehabilitation, motor axonal neuropathy, op poisoning

## Abstract

In India, organophosphorus (OP) chemicals known as anticholinesterases cause a considerable amount of disease and mortality. While precise figures are unavailable, data from hospitals indicates that about 50% of acute poisoning episodes are attributed to organophosphates. Anticholinesterases, when accidentally or suicidally exposed, cause three different neurological disorders. The first is an acute cholinergic crisis that can be fatal and necessitates administration in an intensive care unit; the second is an intermediate syndrome that frequently results in cranial nerve palsies, proximal and respiratory muscle weakness, and respiratory support for patients; and the third is a delayed organophosphate-induced polyneuropathy. Together, these neurobehavioral alterations have been identified and are referred to as "chronic organophosphate-induced neuropsychiatric disorders" (COPIND). A 40-year-old male patient tried suicide by swallowing a significant dose of OP pesticide. He was breathing heavily, gasping for air, foaming at the lips, and smelled intensely of pesticide when he was brought to a private hospital. Investigations like nerve conduction velocity (NCV) were done, which revealed motor axonal polyneuropathy.

## Introduction

Organophosphorus (OP) compounds are widely used in agriculture as pesticides and in various industrial processes. Despite their effectiveness in controlling pests, exposure to these compounds can lead to severe health consequences, particularly when poisoning occurs. One of the detrimental effects of OP poisoning is motor axonal neuropathy, a condition that affects the peripheral nervous system and impairs motor function. The introduction of the intermediate condition, symptoms, and treatments associated with both acute and chronic exposure to OP are briefly mentioned. There are recommendations made for reducing the acute and long-term effects of OP consumption both locally and systemically [1]. About 75% of OP is metabolized during metabolic into metabolites known as dialkyl-phosphate metabolites, which, at high dosages, can be somewhat neurotoxic [2]. By blocking acetylcholinesterase in the central and peripheral nervous systems, OPs function as neurotoxins, causing an excess of acetylcholine (ACh) and a variety of neurological symptoms [3].

OP affects a developing fetus's and child's neurobehavioral development even at extremely low exposure levels. Male and female infertility, as well as testicular cancer, have been related to long-term exposure to OP. A central-distal axonopathy, organophosphate-induced delayed neuropathy progresses via latent, progressing, static, and improving stages. The spinal cord lesion with myelopathic characteristics is shown by the regeneration of peripheral nerves during the improvement phase. This case study aims to explore the crucial role of physical rehabilitation in managing motor axonal neuropathy following organophosphorus poisoning. Motor axonal neuropathy is characterized by damage to the axons of motor neurons, leading to muscle weakness, atrophy, and impaired motor coordination. The severity of symptoms can vary, ranging from mild weakness to complete paralysis [4].

Acute organophosphate exposure

Emergency rooms (EDs), critical care units, and medical wards frequently witness suicide attempts via over-the-counter (OP) medications in global farming regions, particularly those where gun ownership is forbidden [5]. Acute OP poisoning manifests as a quick start of symptoms after cutaneous absorption, inhalation, oral ingestion, or injections; the degree of symptoms depends on the particular chemical, quantity, exposure method, and rate of metabolism breakdown [6].

Immediate or severe neurological and respiratory crisis is the outcome of acute OP exposure. Because of how the substance interacts with muscarinic and nicotinic receptors as well as the central nervous system, OP intoxication is linked to increased morbidity and death [1,7]. In cases of OP poisoning in children, seizures are more common than in adult cases and may provide a diagnostic signal in the early differential diagnosis. Following OP poisoning, the three stages of neurological disease are widely documented. In cases of OP poisoning in children, seizures are more common than in adult cases and may provide a diagnostic signal in the prompt alternative diagnosis. Following OP poisoning, the three stages of neurological disease are widely documented [8]. Two to three weeks following OP exposure, phase III (organophosphate-induced delayed polyneuropathy) manifests. Either recurrent or individual encounters with OP chemicals have several harmful impacts on the health of people [9].

Chronic organophosphate exposure

Healthcare personnel need to prevent infecting their own when treating individuals with OP poisoning. Safety attire (e.g., rubber gloves and gowns) is required while disinfecting those suffering from OP poisoning since hydrocarbons may permeate non-polar material like latex and vinyl. Detoxifying treatments are used for managing the long-term consequences of poisonous substances. Consuming enough amounts of docosahexaenoic acid, a form of omega-3 fatty acid derived from marine life that includes sardines, herring, and sardines, can serve to boost antioxidant metabolism in neurons to avoid organophosphate-induced damage [10]. Antioxidants such as vitamin E, vitamin C, and alpha-lipoic acid may potentially prevent organophosphate-induced cellular damage [11,12].

## Case presentation

Patient information

A 40-year-old male patient attempted suicide by taking a big dose of OP pesticide. He was taken to a private hospital unconsciously, breathing, foaming at the mouth, and smelled of pesticide. Pupils were miotic, and fasciculations were seen while on synchronized intermittent mandatory ventilation (SIMV), and he was managed conservatively in a local hospital, but the patient symptoms were not recovered. Then, he was referred to Acharya Vinoba Bhave Rural Hospital (AVBRH), Sawangi, Wardha, casualty, where the patient was intubated (tracheostomized) and admitted to the intensive care unit for further investigation and nerve conduction velocity (NCV) was done, which revealed motor axonal polyneuropathy. Then, he was referred to the Neuro Physiotherapy unit.

Clinical findings

On neurological examination, the patient was conscious and disoriented. Prior to beginning the physical exam, the patient provided consent via speech. He was assessed in a supine lying position. Upon inspection, it was observed that the patient was supine, lying with the head end elevated to 30 degrees. The patient’s hands and legs were extended and slightly abducted. Ryle’s tube was present. On examination, the Glasgow Coma Scale (GCS) score was 11/15 on the day of assessment (E4VTM6). His blood pressure was 120/80 mm Hg, pulse rate was 80 beats/minute, respiratory rate was 24 breaths/minute, body temperature was 37°C, and he was maintaining oxygen saturation in room air. On palpation, the local skin temperature was raised. The timeline of events is shown in Table 1, muscle tone is shown in Table 2, and manual muscle testing (MMT) is shown in Table 3. On sensory examination, the patient responds to painful stimuli.

**Table 1:** Timeline of events AVBRH: Acharya Vinoba Bhave Rural Hospital, OP: organophosphorus.

Incidents	Dates
Consumption of OP poisoning	05/11/2023
Date of admission in AVBRH	05/11/2023
Date of physiotherapy commencement	21/11/2023

**Table 2:** Muscle tone of upper and lower extremities 1+: decreased tone; 2+: normal tone.

Muscle tone	Right side	Left side
Upper limb	2+	2+
Lower limb	1+	1+

**Table 3:** Manual muscle testing MMT: manual muscle testing, 3: active movement against gravity, 4: active movement against some resistance, 5: active movement against maximum resistance.

MMT	Right side	Left side
Upper limb	3/5	4/5
Lower limb	3/5	3/5

Diagnostic assessment

**Figure 1:**
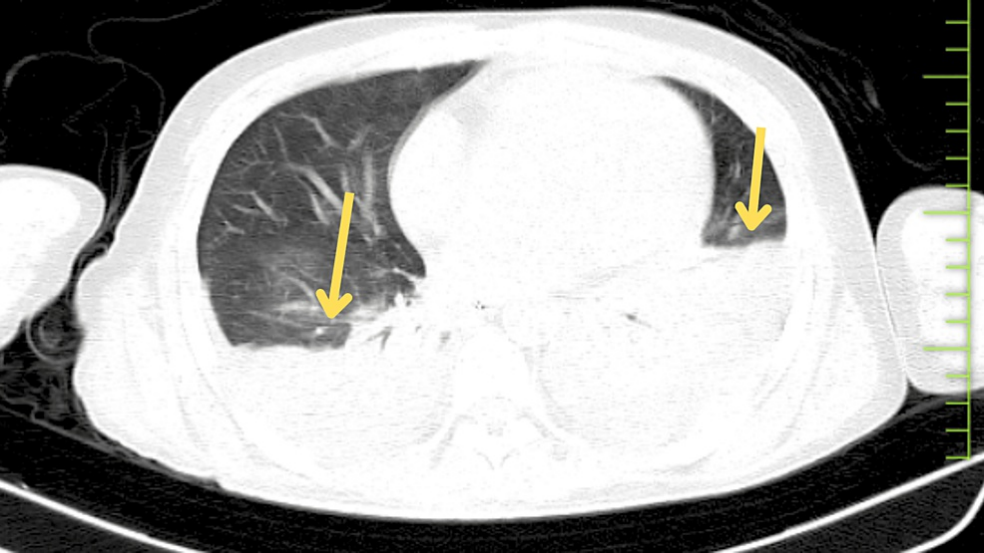
HRCT thorax HRCT: high-resolution computed tomography. Yellow arrow points toward bilateral pleural effusion (left>right) with subsegmental atelectasis.

NCV reports revealed motor axonal polyneuropathy, and high-resolution computed tomography (HRCT) thorax reports revealed bilateral pleural effusion (left>right) with subsegmental atelectasis (shown in Figure 1).

Therapeutic management

**Figure 2:**
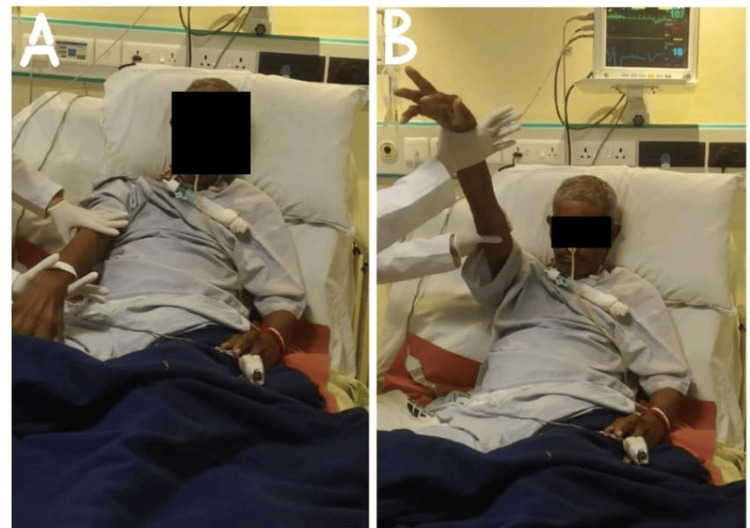
Active-assisted ROM of right upper limb ROM: range of motion. A: The patient initiated the ROM of the right-side upper limb. B: The patient preform active-assisted ROM of the right-side upper limb.

**Figure 3:**
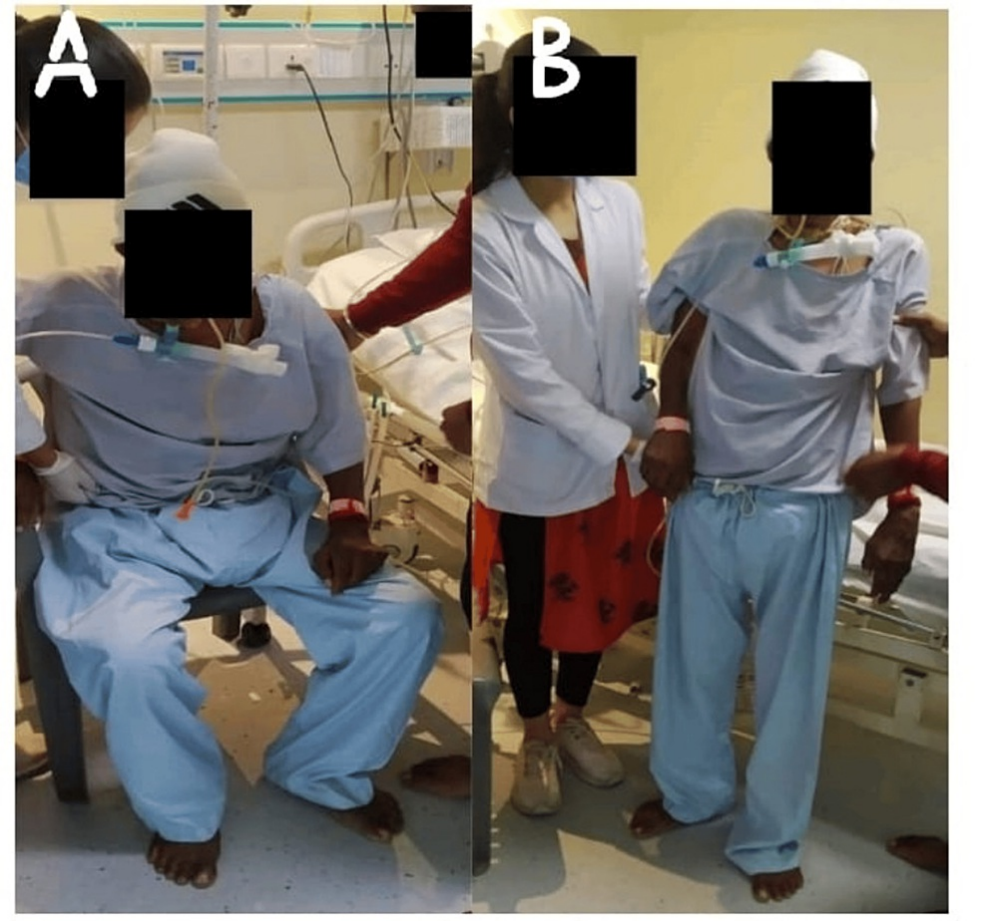
Sit to stand with moderated assistance A: The patient was in sitting position. B: The patient was able to stand with moderated assistance.

The goal-oriented physiotherapy protocol is given in Table 4. The range of motion of the upper limb is shown in Figures 2A, 2B in which the patient preform active-assisted range of motion. The patient preform sit-to-stand activity with moderated assistance is shown in Figures 3A, 3B. Pre- and post-rehabilitation outcome measures and follow-up are mentioned in Table 5.

**Table 4:** Therapeutic management ROM: range of motion, PNF: proprioceptive neuromuscular facilitation, D1: diagonal pattern.

Goals	Therapeutic intervention	Treatment protocol
Patient education	A patient is informed about their disease as well as the importance and advantages of physical rehabilitation, obtaining the family members' permission and gaining confidence.	Caregivers were educated about the importance of timely body positioning.
To improve ROM of the upper and lower limbs	Active-assisted ROM exercises to the right upper limb and lower limb. Passive ROM exercises to the left upper and lower limbs.	A double set of 10 repetitions two times each day.
To normalize muscle tone	PNF rhythmic initiation D1 flexion-extension to the left upper and lower limbs. Joint approximation to the upper limb and lower limbs.	A double set of 10 repetitions two times each day.
To ensure good ventilation	Deep breathing exercises and pursed lip breathing exercises.	A single set of ten repetitions, two times each day.
Improve airway clearance	Positioning, manual chest techniques (percussion and vibrations), and suctioning	Changing positions every two to three hours and manual methods for 10 repetitions.
Bed mobility	Supine, side lying, bed side sitting with maximal assistance.	2 times a day for 1 week.
To avoid pressure sores resulting from extended immobilization	Manual positioning and air bed provided.	Positioning for every 2-3 hours.
Difficulty in walking	Maintain standing balance. Gait training	Standing within parallel bars. Initiate with spot marching and progress to walking between the bars. Progression to tandem walking, side walking, walking between obstructions, and step training.
Getting out of bed with a wheelchair	To encourage the patient's mobility and raise their level of attentiveness.	Every day for 2 hours.

Follow-up and outcome measure

An organized physical therapy interventional protocol was started. For four weeks, a follow-up was carried out once per week. The findings of the outcome measure before and after physiotherapy sessions are shown in (Table 5) 

**Table 5:** Follow-up and outcome measure ICU: intensive care unit.

Sr.no	Outcomes measure	Pre-physical therapy rehabilitation	Post-physical therapy rehabilitation
1.	Glasgow Coma Scale	11/15	13/15
2.	Tone grading scale	1+	2+
3.	Functional independence scale	1/7	6/7
4.	ICU mobility score	1/10	7/10

## Discussion

This case study aims to explore the crucial role of physical rehabilitation in managing motor axonal neuropathy following organophosphorus poisoning. Motor axonal neuropathy is characterized by damage to the axons of motor neurons, leading to muscle weakness, atrophy, and impaired motor coordination. The severity of symptoms can vary, ranging from mild weakness to complete paralysis. The rehabilitation process involves a multidisciplinary team, including physiotherapists, occupational therapists, and other healthcare professionals. These experts collaborate to design individualized rehabilitation programs tailored to the specific needs and challenges faced by patients with motor axonal neuropathy. The interventions aim to address muscle weakness, improve mobility, and enhance overall physical function. While the immediate treatment of organophosphorus poisoning often involves the administration of antidotes and supportive care, the long-term consequences, especially motor axonal neuropathy, necessitate a comprehensive rehabilitation approach. Physical rehabilitation plays a vital role in promoting recovery, improving functional outcomes, and enhancing the quality of life for individuals affected by this condition.

Organophosphate esters find applications as pesticides, flame-retardants, lubricants, petroleum additives, and plastic modifiers. They can enter the body through the cutaneous, gastrointestinal, or respiratory systems [13]. The ability to penetrate both the central and peripheral nerve systems is facilitated by liposolubility [14]. Each has distinct characteristics and a distinct time frame for occurring after consuming an organophosphorus compound (OPC). Intermediate syndrome (IMS) manifests one to four days following consumption and is accompanied by cranial nerve involvement and weakness in the proximal, neck, and extraocular muscles. The electromyography (EMG) displays tetanic fading. Two to three weeks after ingesting OPC, distal muscular weakness, with or without sensory involvement, is the clinical manifestation of organophosphate-induced neuropsychiatric disorder (OPIND). Potentiation of denervation is seen in EMG. Between four and 40 days, chronic organophosphate-induced neuropsychiatric disorders (COPIND) may show up as extrapyramidal and psychological signs [15].

One rare cause of polyneuropathy (PNP) is OPIND. Reviewing the history of exposure to harmful chemicals is crucial when looking into the causes of peripheral neuropathy. While the immediate treatment of organophosphorus poisoning often involves the administration of antidotes and supportive care, the long-term consequences, especially motor axonal neuropathy, necessitate a comprehensive rehabilitation approach. Physical rehabilitation plays a vital role in promoting recovery, improving functional outcomes, and enhancing the quality of life for individuals affected by this condition.

The patient then experiences IMS as a result of the impact on the nicotinic receptor. This patient's symptoms were cranial nerve palsy, respiratory exhaustion or failure, and leg and neck muscular weakness. This stage required mechanical ventilation for a brief period. Later, he had OPIND, which was characterized by paresthesia, motor weakness, cramping discomfort in the muscles, and the absence of deep tendon reflexes with normal joint position and vibration perception. After six weeks of poisoning, our patient's corticospinal tract symptoms gradually improved, exhibiting aberrant posture, stiffness, and heightened deep tendon reflexes. We call this OPIND [16]. Long-term care is required for OPIDN, and physiotherapy is crucial to the patient's ability to operate independently and integrate into the community. This implies that choosing the right physiotherapy modality at different stages requires constant assessment of changes in indicators. To maintain the patient's functional integrity, rehabilitation techniques must be devised before the patient is discharged, and ongoing follow-up calls for constant contact between the patient and a community rehabilitation physiotherapist [17].

## Conclusions

When certain OP esters are exposed, an uncommon toxicity known as OPIND can occur. It is typified by one to four weeks following a single or brief exposure to certain peripheral and central nervous system axons that have undergone distal degeneration. It is thought to be caused by the phosphorylation and aging of neurotoxic esterase, also known as neuropathic target esterase (NTE), an enzyme found in axons. The use of anticholinergic drugs for the cholinergic crisis and the chemical structure and dosage of the organophosphates are two important elements that affect the long-term neurotoxicity caused by organophosphorus poisoning. The goal of physical treatment was to avoid neuro-musculoskeletal co-morbidity. The paralyzed muscles of the upper and lower extremities were stimulated electrically using intermittent galvanic current in order to restore their characteristics and stop further muscle loss. Further abnormalities were averted with hand flexor and gastro-soleus stretching, proprioceptive neuromuscular facilitation for weak muscles, and orthotic devices. The negative effects of extended bed rest and postural hypotension were mitigated with the use of tilt table-aided standing. It has a big impact on improving patients' quality of life and helping them restore their physical abilities.
